# Health-related quality of life in adults undergoing transthoracic device closure of ventricular septal defect

**DOI:** 10.1186/s13019-019-1004-x

**Published:** 2019-10-21

**Authors:** Kai-Peng Sun, Qiang Chen, Zhi-Nuan Hong, Jiang-Shan Huang, Hua Cao

**Affiliations:** 1 Department of Cardiac Surgery, Fujian Provincial Maternity and Children’s Hospital, affiliated Hospital of Fujian Medical University, the Daoshan Road 18, Gulou District, Fuzhou, 350001 People’s Republic of China; 2 Department of Cardiovascular Surgery, Union Hospital, Fujian Medical University, Fuzhou, 350001 People’s Republic of China

**Keywords:** Health-related quality of life, Ventricular septal defect, Hybrid procedure

## Abstract

**Objective:**

To evaluate the health-related quality of life (HRQoL) of adult patients who underwent transthoracic device closure of ventricular septal defect (VSD).

**Methods:**

During the perioperative and postoperative period, a standard scale involving eight dimensions was used to analyze the HRQoL of 85 adult patients who underwent thoracic device closure of VSD and 80 healthy adults located locally were randomly selected as the control group in our center.

**Results:**

A total of 80 patients’ and 80 healthy adults’ questionnaires were received with complete feedback. Out of all of the items that were investigated, postoperative patients experienced better feelings in some dimensions than the control group. Postoperative feedback was also better than preoperative feedback in some dimensions. In the comparison of the subgroups of these patients, the scores of the elderly were lower than those of the young in most dimensions. Males had more positive feedback in two aspects (“role-physical” (*p* = 0.01) and “vitality” (*p* = 0.003)), whereas unmarried people seemed to have poor emotional responses (“role-emotional” (*p* < 0.01) and “vitality” (*p* = 0.023)). There was no significant difference in any dimensions except “social functioning” (p < 0.01) between people with different levels of education.

**Conclusions:**

Most of the adult patients who underwent thoracic closure of VSD felt that they could lead a normal life. They seemed to have reasonably normal psychosocial responses compared to healthy controls. Many patients even though their HRQoL was better than healthy individuals.

## Introduction

In recent years, transthoracic device closure has been widely used in the treatment of ventricular septal defects (VSDs); this procedure has the advantages of no X-ray exposure, easiness to learn and master, and a minimal incision. The indications include clinically significant left-to-right shunts, isolated VSDs, and a VSD size ranging from 4 to 12 mm; exclusions include nonrestrictive or misaligned VSDs, large VSDs, and the presence of severe pulmonary hypertension, a proximal aortic valve, or coexisting cardiac anomalies [1–3]. With the development of such a procedure, the long-term survival rate after transthoracic device closure of VSD in adults is increasing [2]. Numerous studies have focused on the technique’s recent effects and procedural-related complications, such as residual fistula, aortic valve regurgitation, and subsequent complete atrioventricular block [1–3]. Many previous study also confirmed that there are no significant differences in clinical outcomes for VSD between surgical repair and transthoracic device closure [1, 3]. However, no articles have focused on postoperative health-related quality of life (HRQoL) in these patients. HRQoL is a subjective evaluation of patients’ lives rather than an outcome exclusively determined by laboratory test results or functions. The way patients deal with problems caused by related diseases will affect their HRQoL. Therefore, HRQoL is affected by both external variables and individual decisions [4, 5]. In this study, HRQoL was used to evaluate adult patients who underwent transthoracic device closure of VSD in multiple dimensions.

## Methods

From January 2012 to February 2017, 85 adult patients who successfully underwent transthoracic device closure of VSD were interviewed in the outpatient department of our cardiac center. Only adult patients who can agree and willing to complete the questionnaire survey were included in this study, other patients were excluded from this study in the same period. These patients underwent preoperative medical examination and were diagnosed as a simple VSD and no other serious disease. A review of the interview process indicated that the success of the interview was typically dependent on the patient’s personality and willingness to complete the survey. We interviewed all 85 patients who were asked to complete a questionnaire within the preoperative 1 day and the postoperative 1-year’ follow up. The matching of the control group was based on age, gender, marital status, and education level. According to these requirement, 80 healthy local adults were randomly selected from the local physical examination department and their evaluations were also conducted twice over a period of 1 year. These people had a regular physical examination and were certified to be in good health without other serious illnesses or major surgery, such as cardiovascular disease, stomach cancer or kidney dysfunction. Participation was voluntary, and there were no further selection criteria.

### Questionnaire

This study was approved by the ethics committee of Fujian Medical University (#2009024), China, and written informed consent was also obtained from the patients. All the patients and healthy adults completed the questionnaire independently. Afterward, the questionnaires were returned to the research center for further unified evaluation. In the first part, the situation of the patients and the procedurally relevant information about the transthoracic device closure of VSD were investigated by the questionnaire. In the second part, the Medical Outcome Study Short Form 36 (SF-36) was used for HRQoL evaluation. The preoperative and postoperative scores of the patients were compared, and the scores of the patient group and the control group were also compared. Simultaneously, we also compared the postoperative scores of subgroups stratified by age, gender, marital status, and education level.

### Transthoracic device closure of VSD

The procedure was performed under general anesthesia. A lower partial median incision was performed, and the sternum was partially cut. The pericardium was opened and suspended to expose the right ventricle. A puncture was made at the right ventricle, and the guidewire was inserted through the puncture site. With the guidance of transesophageal echocardiography, the guidewire was advanced through the VSD into the left ventricle. The sheath was inserted along the guidewire to establish the transport track. The occluder was implanted along the sheath and released into the two sides of the ventricular septum to close the VSD [1].

### Statistics

SPSS 18.0 was used for statistical analysis. The normality test revealed that not all of the data exhibited a normal distribution. Nonparametric tests were selected for the statistical analysis. The Mann-Whitney U test was used to compare the gender, marital status, case group, and control group. The Kruskal-Wallis H (K) test was used to compare the education background and age group. The Wilcoxon (W) test was used to compare the preoperative and postoperative groups. *P*-values< 0.05 indicated significant differences between groups.

## Results

### Research respondents

Of the 85 patients interviewed, 80 questionnaires were completed and returned for further evaluation. There were 48 males and 32 females. In total, 35 patients were between 18 and 30 years, 37 were between 31 and 50 years, and eight were older than 50 years. Fifty patients were married, and 30 were unmarried. Twenty-five patients had an undergraduate degree or higher, 40 had a high school diploma, and 15 had a junior high school education or below. Of the 80 healthy adults interviewed, there were 44 males (55.0%) and 36 females (45.0%). In total, 32 respondents were between 18 and 30 years, 38 were between 31 and 50 years, and ten were older than 50 years. Fifty-five respondents were married, and 25 were unmarried. Thirty-two respondents had an undergraduate degree or higher, 34 had a high school diploma, and 14 had a junior high school education or below. By comparing the demographic data between the control group and the case group, it was found that there was no significant difference between the two groups in age, gender, marriage status and education level.(Table. 1).

**Table 1:** Demographics and clinical characteristics of the participants

Item		Case group	Control group	P
Age (years)	18–30	35 (43.8%)	32 (40.0%)	0.823
	31–50	37 (46.2%)	38 (47.5%)	
	> 50	8 (10.0%)	10 (12.5%)	
gender	Male	48 (60.0%)	44 (55.0%)	0.764
	Female	32 (40.0%)	36 (45.0%)	
Marital status	married	50 (62.5%)	55 (68.8%)	0.852
	Unmarried	30 (37.5%)	25 (31.2%)	
Education	Junior high school or lower	15 (18.8%)	14 (17.5%)	0.926
	High school	40 (50.0%)	34 (42.5%)	
	bachelor’s degree or higher	25 (31.2%)	32 (40.0%)	
Height (cm)		170.3 ± 10.4	168.8 ± 11.2	0.873
Body mass (kg)		67.2 ± 10.8	64.8 ± 9.5	0.931
Heart rate		81 ± 14.1	78 ± 13.2	0.852
LVEF (%)		53.5 ± 5.2	65 ± 4.0	0.756

Data are presented as mean ± standard deviation or percentage

*LVEF* left ventricular ejection fraction

### Preoperative and postoperative comparison

**Fig. 1:**
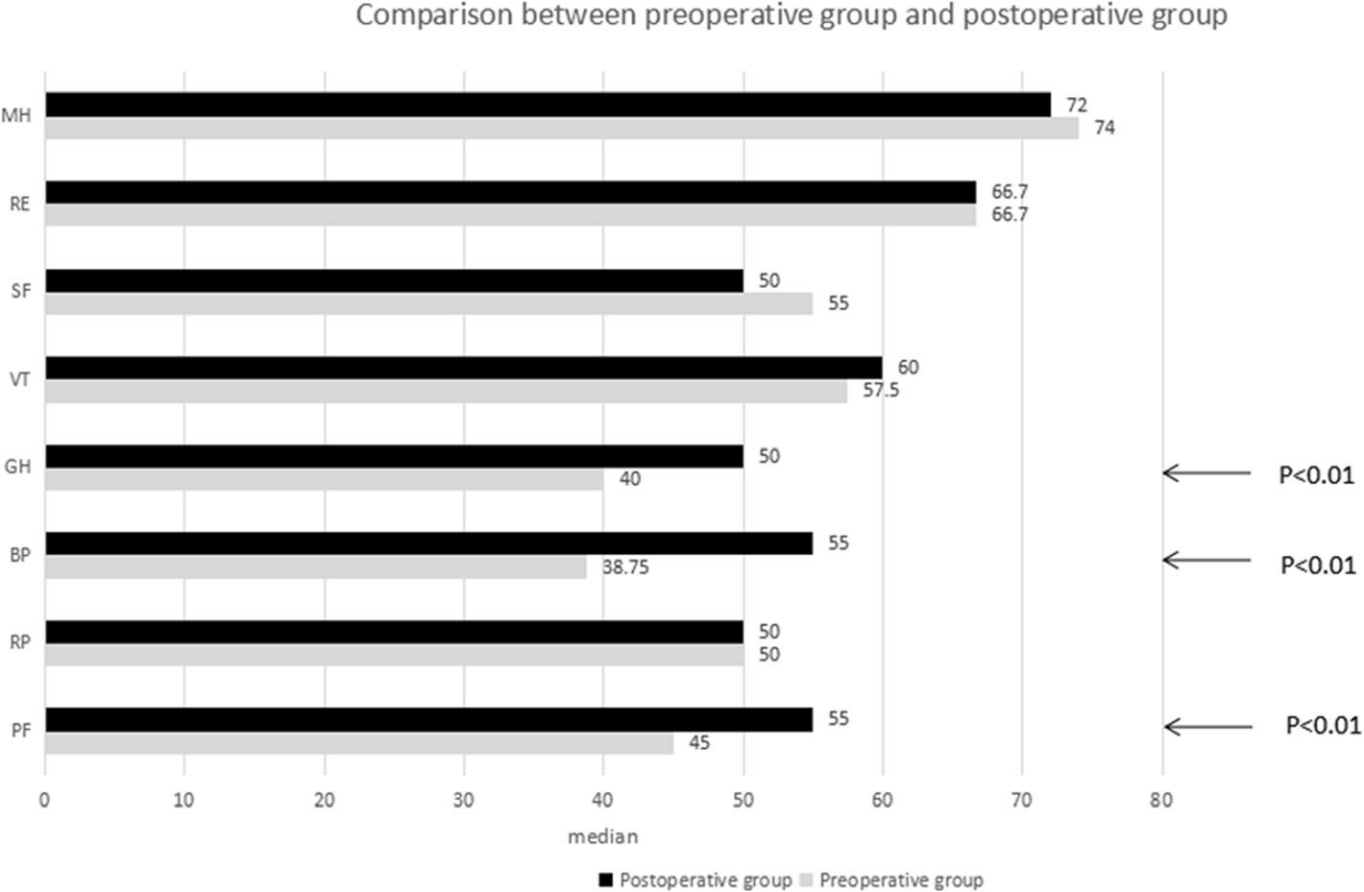
Median of the ranking of quality of life in patients after transthoracic closure of VSD in adults. Comparison of preoperative and postoperative patients. A high value indicates a positive ranking

By comparing the patient’s SF-36 scores in all dimensions before and after the procedure, it was found that the postoperative scores were improved for “bodily pain” (*p* < 0.01), “physical functioning” (p < 0.01), and “general health” (p < 0.01) compared with those before the procedure, and no significant difference was noted in the other dimensions (Fig. 1).

### Comparison between the case group and the control group

**Fig. 2:**
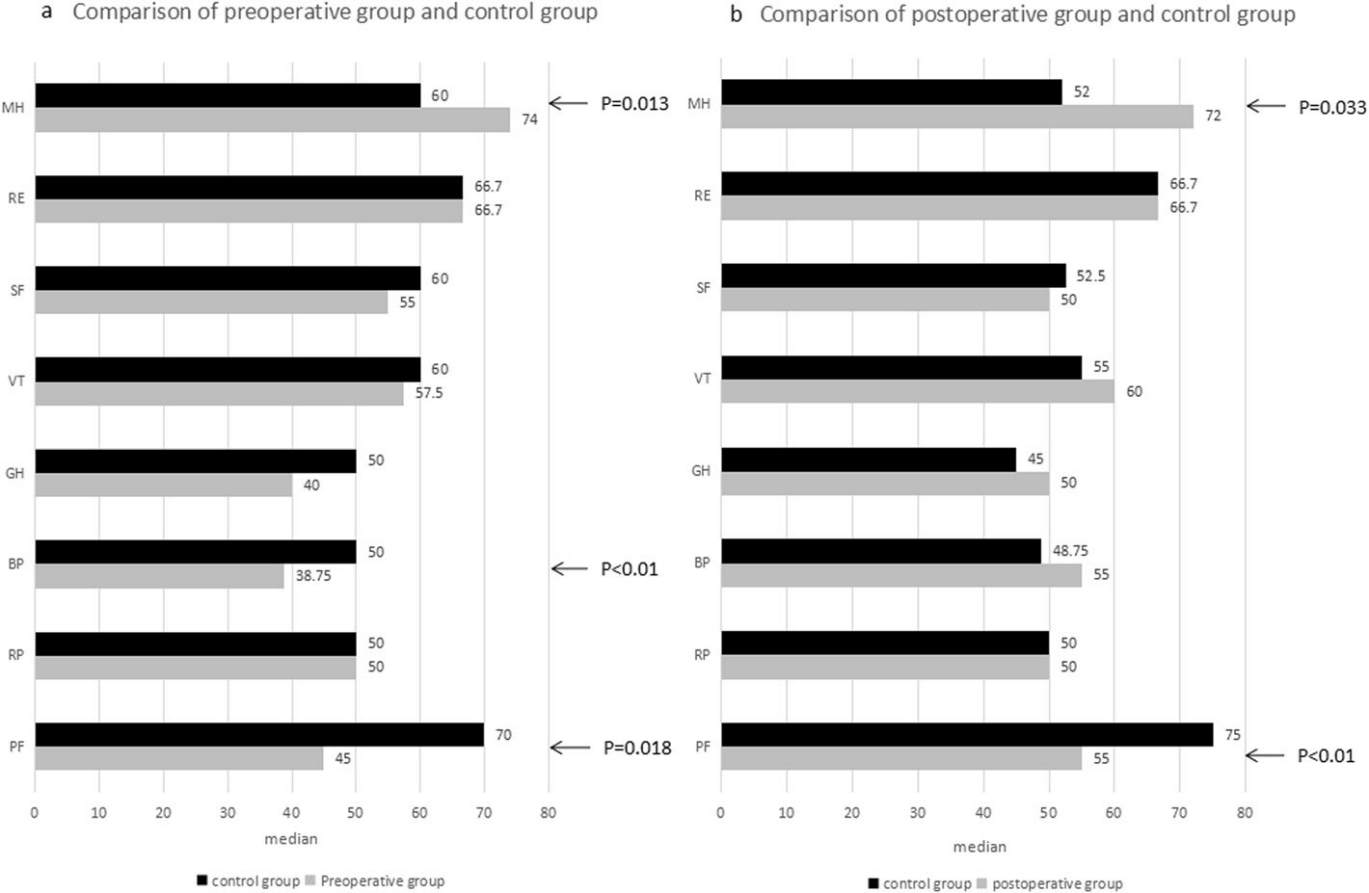
**a** Median of the ranking of quality of life in patients before transthoracic closure of VSD in adults compared to that of healthy controls. A high value indicates a positive ranking. **b** Median of the ranking of quality of life in patients after transthoracic closure of VSD in adults compared to that of healthy controls. A high value indicates a positive ranking

After comparing the SF-36 scores of the preoperative case group and the control group, it was found that the scores of the control group were better than those of the preoperative case group in two dimensions of “bodily pain”(*p* < 0.01) and “physical functioning”(*p* = 0.018). In terms of “mental health”(*p* = 0.013), the preoperative case group was better than the control group, and no significant difference was noted in the other dimensions (Fig. 2a).

HRQoL feedback in the postoperative case group was superior to that of the control group in most dimensions. In particular, scores for “mental health” (*p* = 0.033) were significantly higher than those of the control group, and in the field of “physical functioning” (*p* < 0.01), the score of the postoperative case group was lower than that of the control group (Fig. 2b).

### Case subgroup internal comparison

**Fig. 3:**
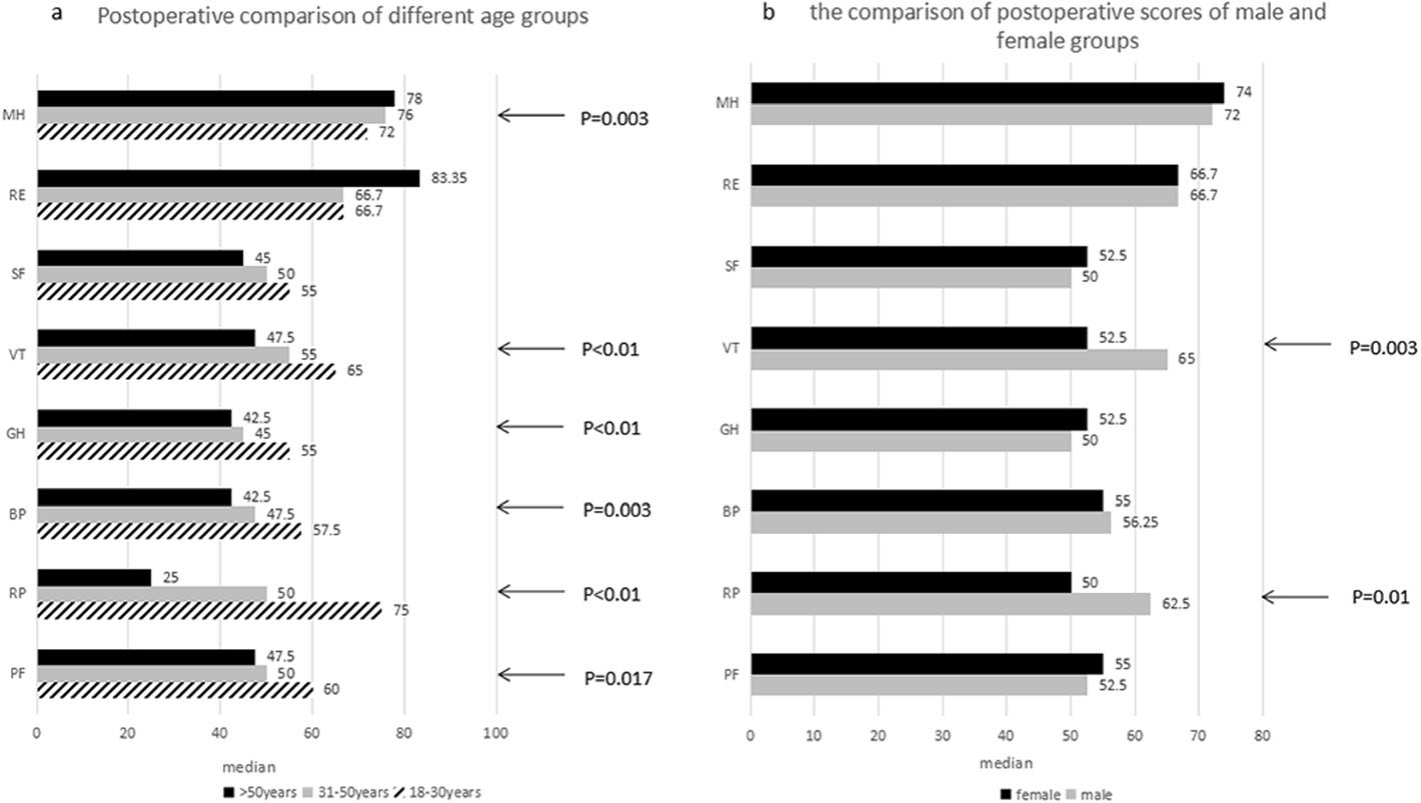
**a** Median of the ranking of the quality of life in patients after transthoracic closure of VSD in adults according to age group. A high value indicates a positive ranking. **b** Median of the ranking of quality of life in patients after transthoracic closure of VSD in adults (comparison of 42 male vs. 38 female patients). A high value indicates a positive ranking

Comparisons between different age groups were made in the case group. In addition to “role-emotional” and “mental health” (*p* = 0.003), scores for most items tended to decline with age. In particular, the scores of patients over 50 years old in the three dimensions of “role-physical” (*p* < 0.01), “vitality” (p < 0.01) and “bodily pain” (*p* = 0.003) were significantly lower than those of the younger patients, whereas the scores of the older patients in the fields of “role-emotional” and “mental health” (p = 0.003) were more positive (Fig. 3a).

In the comparison between males and females, no significant gender differences in scores were noted for most dimensions, with the exception that males were more positive in the fields of “role-physical” (*p* = 0.01) and “vitality” (p = 0.003) (Fig. 3b).

**Fig. 4:**
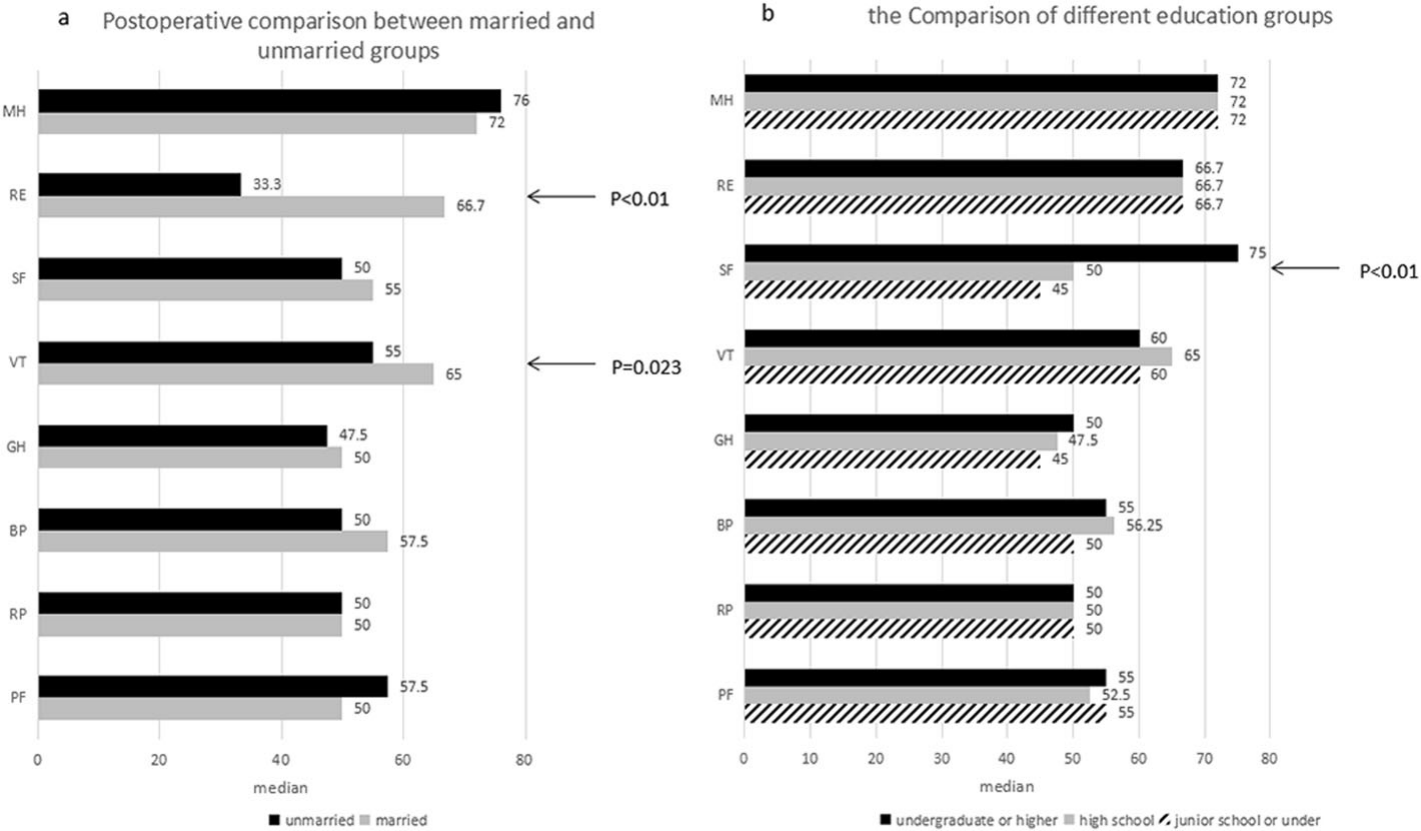
**a** Median of the ranking of quality of life in patients after transthoracic closure of VSD in adults (comparison of 50 married vs. 30 unmarried patients). A high value indicates a positive ranking. **b** Median of the ranking of quality of life in patients after transthoracic closure of VSD in adults according to different education statuses. A high value indicates a positive ranking

In the comparison between the married group and the single group, except for the fields of “role-emotional” (*p* < 0.01) and “vitality” (*p* = 0.023) in which the married group was more positive, no significant differences were noted between the married group and the single group in other fields (Fig. 4a).

In the comparison between different education level groups, except for the score of “social functioning” (p < 0.01) that increased as the educational level increased, no significant differences were noted in the other dimensions (Fig. 4b).

## Discussion

VSD is one of the most common congenital heart diseases, and 70% of cases are perimembranous VSD [6]. Surgical treatment is considered to be an effective method for all types of VSD. Among the many surgical modalities for VSDs, open-heart repair with median sternotomy is the mainstream therapy, whereas minimally invasive partial sternotomy and right minithoracotomy are used to reduce trauma and increase cosmetic results [7, 8]. Transcatheter device closure of VSD has been used because it produces less scarring, but the procedure is still associated with X-ray exposure [9, 10]. Recently, minimally invasive transthoracic device closure of VSD has been widely used in China and has achieved satisfactory clinical outcomes [1–3]. Its advantages include the lack of a need for cardiopulmonary bypass, no exposure to X-rays, and a small incision in the chest. The procedure can be easily converted to surgical repair immediately upon failure of the device closure. Adult patients with VSD can be completely anatomically corrected and survive for a long time by choosing a different closing method. In most adult cases subject to device closure of VSD, no complications are associated with the procedure, and the patient’s physical condition is improved compared with that before the procedure [2]. Given that adult patients have complete behavioral and cognitive abilities, we should also pay attention to the patient’s quality of life after VSD device closure as well as the disease and treatment process. It is also an essential part of the search for such a disease.

HRQoL is commonly used as a prognostic indicator. It is based on the standard of living and mainly focuses on the assessment of the degree of satisfaction people experience regarding their spiritual and cultural needs and environmental conditions. The connotation of HRQoL has great universality and integrity, which includes psychological, physical and social functioning. HRQoL measurements are increasingly seen as useful indicators of health outcomes and the effectiveness of health services [11]. Postoperative pain, psychological changes, and other factors affect a patient’s quality of life, and the quality of life may also be affected by various aspects of daily life, such as interpersonal communication, sports, life rhythms, etc. [12, 13] Reduced cardiopulmonary and psychological function may also contribute to a reduced quality of life. In addition, quality of life is also influenced by numerous other factors, such as economic situation and emotional experience [14, 15].

The SF-36 questionnaire used in this study is a standardized questionnaire that is now widely used for both research and clinical purposes, and it has undergone rigorous psychometric evaluation nationally and internationally using classical test theory (CTT) [16–20]. As a general questionnaire, it includes the following eight different areas: 1. physical functioning (PF); 2. role-physical (RP); 3. bodily pain (BP); 4. general health (GH); 5. vitality (VT); 6. social functioning (SF); 7. role-emotional (RE); and 8. mental health (MH). Different questions explore each field, and each question corresponds to the different frequency levels of answers and corresponding scores. Respondents must choose one response as the answer. PF, RP, BP, and GH comprise the physiological field, whereas VT, SF, RE, and MH comprise the psychological field [21]. Due to its extensive use in patients and the general population, the SF-36 general questionnaire also allows for the comparison of patients’ health status with a variety of diseases and in comparison to that of the general population [22]. In this study, the preoperative and postoperative quality of life of 85 patients who had undergone transthoracic device closure of VSD were investigated, and 80 patients completed the investigation. Of these 80 patients, slightly more men than women responded (40.0% female vs. 60.0% male). In addition, 62.5% were married, and the education level of these 80 patients was mainly high school (50.0%). Among the five patients who failed to complete the investigation, three were unable to complete the investigation due to lack of sufficient time, and two were unwilling to cooperate with the investigation.

After analyzing the data, the physiological field score was more improved for the postoperative data of the patients than for the preoperative data. The reason may be that after device closure of VSD, the patient has a subjective sense of his/her physical condition that it is better than before the procedure [23]. In the comparison between the preoperative case group and control group, the score of the case group was similar to that of the control group in many dimensions, but in terms of “bodily pain”, the preoperative case group was significantly different from the control group. After all, patients suffer from a long-term illness, which has a physical and psychological impact on them, making them less tolerant of pain than the general population. And In the comparison between the postoperative case group and control group, the HRQoL of adult patients with transthoracic device closure of VSD was higher than that of the control group in many dimensions, especially in terms of psychological construction. This may be because the patient’s mental stress was significantly reduced, and the ability to bear pressure and the desire for life was significantly improved. However, the scores in the “physical functioning” dimension were lower in the patient group than in the control group. This finding may be related to the psychological state of patients who felt that their physical condition after the procedure was not as good as that of healthy people [24, 25].

After the statistical analysis of different subgroups, the following results were observed: 1. The quality of life (PF, RP, BP, GH, VT, MH scores) of patients with transthoracic device closure of VSD at different ages was significantly different. The environmental field exhibited a negative trend with age, which may be related to the natural tendency that an individual’s physical fitness declines as he/she gets older. In the dimensions of “role-emotional” and “mental health”, older patients seemed to score better than younger patients possibly because their rich life experience enabled them to face disease more calmly [26]. 2. The scores in the dimension of “role-physical” and “vitality” of male and female patients was significantly different. In the dimension of “role-physical” and “vitality”, men seemed to score better than women. This finding may be attributed to the notion that men are more passionate about life and more resilient to stress [27, 28]. 3. The quality of life (RE and VT scores) between patients who were married and unmarried was significantly different. Married people seem to have higher quality of life scores, which may be related to the fact that emotional needs are more easily met after marriage. During long-term suffering with a disease, the company of a partner can often reduce the psychological pressure of patients. 4. The quality of life of patients with different educational backgrounds was significantly different; the higher the education, the higher was the quality of life score. This finding may be related to the influence that knowledge and experience acquired by higher education has on patients’ social function. Patients with higher education are more likely to have a clearer understanding of their diseases; consequently, they face their illnesses more calmly. It can be concluded that the quality of life score of adult patients who had undergone transthoracic device closure of VSD is affected by gender, age, marriage status, and education level.

## Limitations

Given that the materials used in this study were limited to adult patients who underwent transthoracic device closure of VSD at one cardiac center, the accuracy of the results may be limited to a certain extent and cannot represent other areas or different races. In addition, since the sample size of this study was only 80 cases, and the sample size of the control group was also 80 cases, multicenter and larger samples must be evaluated to confirm the results. The further research about the comparison of quality of life in adults undergoing transcatheter or transthoracic device closure of VSD should be done in future.

## Conclusion

After preoperative and postoperative evaluations were performed on adult patients who underwent transthoracic device closure of VSD, we found that the patients’ physical experience was improved postoperatively, whereas their psychological experience was not significantly better. Compared with healthy individuals, the patients felt that their quality of life was not much different, and they felt that only their physical functions were slightly worse. The patients also seemed to have reasonably normal psychosocial health, and some patients even felt better. The HRQoL of adult patients improved and was affected by gender, age, marital status, educational level, and other factors.

## Data Availability

Data sharing not applicable to this article as no data sets were generated or analyzed during the current study.

## Abbreviations

BP: Bodily pain
GH: General health
HRQoL: Health-related quality of life
MH: Mental health
PF: Physical functioning
RE: Role-emotional
RP: Role-physical
SF: Social functioning
SF-36: Medical Outcome Study Short Form 36
VSDs: Ventricular septal defects
VT: Vitality
